# Optimizing structural modeling for a specific protein scaffold: knottins or inhibitor cystine knots

**DOI:** 10.1186/1471-2105-11-535

**Published:** 2010-10-28

**Authors:** Jérôme Gracy, Laurent Chiche

**Affiliations:** 1 CNRS, UMR5048, Université Montpellier 1 et 2, Centre de Biochimie Structurale, 34090 Montpellier, France

## Abstract

**Background:**

Knottins are small, diverse and stable proteins with important drug design potential. They can be classified in 30 families which cover a wide range of sequences (1621 sequenced), three-dimensional structures (155 solved) and functions (> 10). Inter knottin similarity lies mainly between 15% and 40% sequence identity and 1.5 to 4.5 Å backbone deviations although they all share a tightly knotted disulfide core. This important variability is likely to arise from the highly diverse loops which connect the successive knotted cysteines. The prediction of structural models for all knottin sequences would open new directions for the analysis of interaction sites and to provide a better understanding of the structural and functional organization of proteins sharing this scaffold.

**Results:**

We have designed an automated modeling procedure for predicting the three-dimensionnal structure of knottins. The different steps of the homology modeling pipeline were carefully optimized relatively to a test set of knottins with known structures: template selection and alignment, extraction of structural constraints and model building, model evaluation and refinement. After optimization, the accuracy of predicted models was shown to lie between 1.50 and 1.96 Å from native structures at 50% and 10% maximum sequence identity levels, respectively. These average model deviations represent an improvement varying between 0.74 and 1.17 Å over a basic homology modeling derived from a unique template. A database of 1621 structural models for all known knottin sequences was generated and is freely accessible from our web server at http://knottin.cbs.cnrs.fr. Models can also be interactively constructed from any knottin sequence using the structure prediction module Knoter1D3D available from our protein analysis toolkit PAT at http://pat.cbs.cnrs.fr.

**Conclusions:**

This work explores different directions for a systematic homology modeling of a diverse family of protein sequences. In particular, we have shown that the accuracy of the models constructed at a low level of sequence identity can be improved by 1) a careful optimization of the modeling procedure, 2) the combination of multiple structural templates and 3) the use of conserved structural features as modeling restraints.

## Background

**Figure 1:**
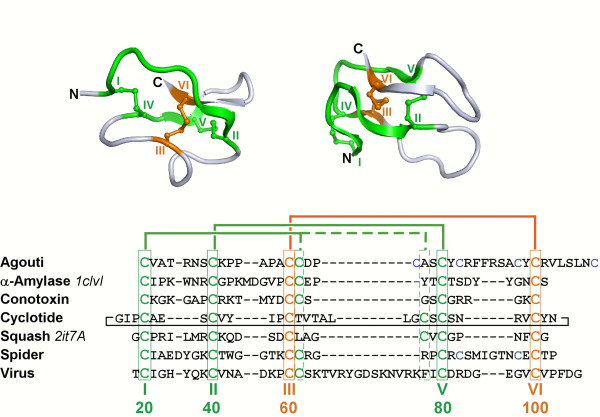
**Top, cartoon representations: 3D structures of the squash inhibitor EETI-II (left, PDB ID **2it7A**) and of the α-amylase inhibitor AAI (right, PDB ID **1clvI). The two-disulfide macrocycle is shown in green and the penetrating disulfide is shown in orange. The structures are in similar but different orientations. Bottom, sequences: selected knottins from 7 families. Families, Swiss-Prot IDs (PDB IDs for the above structures), from top to bottom: Agouti-related, ASIP_HUMAN; α-amylase inhibitor, IAAI_AMAHP (1clvI); Conotoxin1, CXO7C_CONMA; Cyclotide, CYO1_VIOOD, Serine protease inhibitor1, ITR2_ECBEL (2it7A); Spider, TOG4B_AGEAP; Virus1, Q89632_CVS. Knotted cysteines are boxed and numbered at the bottom. Roman numbers indicate the order of knotted cysteines while Arabic numbers indicate standard numbering used in the KNOTTIN database. Cysteine connectivities are shown as thick lines on top of sequences. Sequences were aligned using the Knoter1D tool [1].

The knottin scaffold [1-3] is spread over about 30 distinct disulfide-rich miniprotein families that all share the same special disulfide knot. This knot (Figure 1) is obtained when one disulfide bridge crosses the macrocycle formed by two other disulfides and the interconnecting backbone (disulfide III-VI goes through disulfides I-IV and II-V) [1]. Knottins display a broad spectrum of biological activities and natural members are on the pharmaceutical market or are currently undergoing clinical trials. But knottins also display amazing chemical and proteolytic stabilities, and, thanks to their small size, are amenable to chemical synthesis. Knottins therefore also provide an interesting structural scaffold for engineering new therapeutics and somehow bridge the gap between biological macromolecules and small drug molecules [4,5]. Any such developments, however, would ideally require proper understanding of knottin sequence-structure-function relationships, or at least availability of large sequence and structure data sets. To this goal, we envisaged to extend the KNOTTIN database [1] with quality 3D models of all knottin sequences.

An enormous gap exists between the numbers of sequenced proteins and of solved protein structures (approximately 100 known sequences per structure) and the ratio between the elucidation rates of sequences versus structures tends to increase. To reduce this gap, systematic homology modeling of all proteins with close homologs of known structures has been performed [6-10]. However, the resulting model databases usually do not cover proteins with weakly related structural homologs and these genome wide approaches do not fully exploit all conserved features specific to each protein family as modeling restraints. And indeed, the well-conserved cystine knot which is the main component of all knottin cores should, in principle, facilitate knottin modeling even at very low sequence identity.

Systematically building 3D models for all sequences within a protein family or superfamily could provide additional knowledge for structural or functional analysis and give access to many potential applications [11], but such work has seldom been done [12,13]. Structural models can suggest insight on important residues for protein stability, interaction or function. In particular, the comparison between related protein folds can help to better delineate the key physical and geometrical characteristics of a given interaction site. Such information helps to better understand the mechanisms of molecular interaction and to design focused mutagenesis experiments [14]. Another frequent problem concerns the design of chemical compounds that react selectively with only one type of proteins from the whole family [15]. To this end, if the structures of all homologs of a given protein target are available, the differential analysis of local environments in different model subgroups can help to design highly selective molecules interacting with one subfamily but not with the remaining proteins of the concerned super-family. Homology models can also be useful for the prediction of ligand binding sites [16], for functional annotations [17], or as starting folds for experimental structure determinations [18]. Of course, the best achievable structural model accuracy is critical to extract reliable information from predicted protein folds and give precise answers to the above issues. For this reason, we have optimized a homology modeling method able to systematically predict the fold of all known knottin sequences.

Homology modeling consists in using X-ray or NMR protein structures as templates to predict the conformation of another protein that has a similar amino acid sequence [19]. This structural prediction method has always been the more efficient and rapid way of predicting the folding of a new protein sequence and it should be more and more applicable as fold recognition methods become mature and as the universe of protein folds gets fully covered by experimental structures [20]. Ab initio prediction methods, although achieving spectacular progress in recent years, remain less reliable than homology modeling and are still reserved to proteins that cannot be related to any homologous structure [21,22].

A typical homology modeling of a protein query involves the following processing steps:

1. Identification of query homologs with known structures from the Protein Data Bank [23].

2. Multiple sequence alignment of the query and templates.

3. Construction of structural models satisfying most spatial restraints derived from the query - template alignment.

4. Model refinement.

5. Evaluation and selection of the best model as structural prediction.

The quality of the final 3D models depends on each modeling step and the observed accuracy decreases when the query - template similarity falls down. Homology modeling is efficient because two proteins can have distant sequences but still share very similar folds. But this observation creates also many problems at each step of the modeling when the query and template sequences are weakly similar. A wrong structural template choice might then have a big impact on the query model accuracy. At low sequence identity, query - template alignment is also more ambiguous and any amino acid mismatch will induce important deformations on the resulting structural model. The selection of spatial restraints that should be projected from the templates to the query is another difficult issue when query and templates are only distantly related. In such cases, only a small subset of conserved geometrical features is shared between query and templates, and these can spread over several different structures. Then, insufficient or incompatible spatial restraints extracted from the templates may yield important geometrical variations over the generated models and require further refinement steps such as minimization or loop modeling and accurate structure evaluations to select the best models.

Analyses of known knottin sequences and structures indicate that roughly half of the knottin sequences have to be modeled relatively to weakly related templates. To address this challenge, we have designed a fully automated modeling procedure whose processing steps have been optimized relatively to a test set of 34 known knottin structures. We paid a great attention to the optimal use of the structural information that can be obtained from the available knottin structures. We tried to use the conserved geometrical features derived from the comparative analysis of knottin structures (i) as bias to select templates closer to query, (ii) as anchors to improve sequence alignments, or (iii) as constraints to guide the modeling and increase accuracy. We have tested different structural evaluation methods and designed a combined scoring function for a better assessment of the accuracy of the 3D models. Finally, the models were refined by individual loop modeling and the minimization of the model energy.

## Methods

### Algorithm outline

The structural modeling of a knottin query sequence involves four processing steps:

1. Known knottin structures are sorted according to the similarity of their sequences with the query sequence.

2. The protein query sequence is aligned onto different subsets from the selected knottin templates and is modeled using Modeller [24,25] according to various sequence alignments with the selected knottin templates.

3. The resulting query 3D models are evaluated using various statistical potentials.

4. The best model structure is refined by global minimization of the model energy and individual modeling of each of its loops.

### Test data set

155 knottins with known structures in the Protein Data Bank were extracted from the KNOTTIN database [1]. The quality of these structures was assessed using the program Errat [26] which measures the packing quality of protein structures using atomic-dependent distance statistics derived from the Protein Data Bank [23]. Knottin structures whose Errat scores were below 0.6 were removed from the initial set. Then, to remove data redundancy, the remaining knottin structures were clustered at 40% sequence identity level using the CD-hit software [27]. Within each resulting cluster, the structure with the best Errat score was selected yielding a test set of 34 representative knottin structures.

Each of the 34 selected knottin structures was then modeled from its sequence only at different level of homology using those of the 155 knottin templates which shared respectively less than 10%, 20%, 30%, 40% and 50% sequence identity with the protein query. For example, when the chosen threshold of sequence identity was 30%, no template could share more than 30% sequence identity with the query knottin that should be modelled. In this way, we could evaluate the method performance even at different homology levels, independently of the distribution of the template set.

### Template selection

Three different criteria were tested to select the 3D structures used as templates among the 155 experimental knottin structures for modeling a given knottin query sequence:

1. PID criterion:

The templates were sorted according to their sequence identity percentage relatively to the knottin query sequence.

2. RMS criterion:

This criterion is based on the selection of a reference knottin structure either 1) having the same loop lengths as the protein query, or 2) by default with the highest PID relative to the query.

In the condition 1), the loop lengths are defined as the number of residues of each protein segment between two consecutive knotted cysteines I, II, III, V and VI. The positions of the knotted cysteines and their connecting loops are derived from the purely sequence-based tool Knoter1D [1]. Knoter1D first checks whether the three knotted disulfide bridges are present using an alignment with homologous knottin sequences detected in the annotated KNOTTIN database (http://knottin.cbs.cnrs.fr). Then Knoter1D provides a standard renumbering of each amino acid of the knottin sequence.

In the condition 2), PID is the sequence identity percentage calculated from the comparison of the query and template sequences aligned using CLUSTALW.

Supplementary templates are then selected according to the root mean square deviation of their main chain atoms relatively to this reference knottin structure.

3. DC4 criterion:

Templates were sorted according to the PID criterion less a penalty (-20) if cysteines IV in the template and in the query were not aligned.

### Query - templates alignment

The knottin query sequence was multiply aligned against one or more template structures using two different methods.

1. Alignment method K1D:

The knottin query sequence was aligned using Knoter1D [1].

The knottin template structures were aligned using Knoter3D [1,3]. Knoter3D first searches for the presence of three knotted disulfide bridges from a geometrical analysis of the 3D structure. If this knot is found, the corresponding protein sequence in renumbered such that knotted cysteines I, II, III, V and VI have numbers 20, 40, 60 80 and 100, respectively. It is worth noting that cysteine IV does not get a fixed number as its location changes with families [1,3]. Then the knottin structural core, i.e. the cystine-stabilized beta-sheet (CSB) motif (renumbered residues 40, 60-61, 79-81 and 99-100) [28], is superimposed onto the corresponding motif of a reference knottin structure, from which the optimal structural alignment and its corresponding amino acid numbering is inferred.

Finally, the standard alignment of the knottin query sequence (renumbered using Knoter1D) and of the homologous template sequences (renumbered using Knoter3D) is used for further homologous structural modeling. Detailed descriptions of the Knoter1D and Knoter3D methods can be found in previous publications [1,3].

2. Alignment method TMA:

The 155 knottin templates were globally aligned only once using a hierarchical version of TM-align [29]. All template structure pairs are first aligned using TM-align. Following a decreasing TM-align score order, these template pair alignments were then hierarchically aggregated until all templates were merged into a single multiple sequence alignment. The knotted cysteines that should be aligned are determined by Knoter1D for the query sequence and by Knoter3D for the templates.

Then the query sequence fragment and template profile alignment section located between the N terminus and the first cysteine were multiply aligned using CLUSTALW [30] while keeping the existing indels between templates frozen. This local sequence-profile alignment method was repeated to align the fragments located between the first and second knotted cysteines. This operation was repeated again for all segments connecting the successive knotted cysteines II, III, V and VI. The obtained local alignments were then successively concatenated with the knotted cysteines I, II, III, V then VI in order to obtain a multiple alignment of the query with the templates.

### Model construction

The protein query was modeled multiple times by homology using Modeller [24] through a global alignment of the query with the best template, then with the two best templates, then up to the 20 best templates. The templates were selected using either the PID, RMS or DC4 criterion and aligned with the knottin query using either K1D or TMA method. All known knottin structures were superimposed and hierarchically classified according to their pairwise main chain deviation revealing conserved main chain hydrogen bonds shared by knottins. If more than 80% of the structures of a knottin cluster from the hierarchical tree shared the same hydrogen bond, this bond was said to be "80% conserved". This 80% cut-off was chosen instead of 100% to cope with possible errors or uncertainties in available NMR structures. Five 80% conserved hydrogen bonds were evidenced at standard positions N100-O38, N40-O98, N81-O99, N101-O79 and N79-O101. Four other hydrogen bonds at standard positions N21-O59, N61-O21, N38-O22 and N37-O100 were 80% conserved over the 85 knottin structures with cysteine IV at standard position 61. Standard positions were calculated by the global knottin alignment program Knoter3D [3]. The 3 knotted disulfide bridges and these 80% conserved main chain hydrogen bonds were kept semi rigid by adding geometrical restraints in the Modeller script. At each Modeller run, 1 to 5 different structural models of the protein query were generated. For example, if the maximum allowed number of templates was 20 and if 5 models were generated at each Modeller run, then 5 models were constructed from an alignment with the best template alone, 5 models from the two best templates and so on up to the 20 best templates, resulting in 100 generated models from varying numbers of templates. To remove all minor conformational inconsistencies resulting from the Modeller construction, all models were energy minimized with restraints on the backbone atoms using the Amber package [31].

### Model evaluation

The accuracy of the best selected model was measured by the root mean square deviation (RMSD) between the native and model backbones of the structural segments located between the first and the last knotted cysteines after optimal 3D superposition. When the knottin query corresponded to a PDB entry containing multiple NMR conformers, the first NMR conformation was systematically selected as reference for measuring the model to native structure RMSD.

The similarity between the model and native structure was also assessed using the TM-align score [29] where core conservation is emphasized and long loop moves are scaled down according to the formula:

$$\;\text{TMS}=\text{1}/L.\sum_{i}\text{1}/(\text{1}+{\text{(}D_{i}/D_{0}(L)\text{)}}^{\text{2}})$$

where

*L *is the length of the shortest protein sequence,

*D*_*i *_is the Euclidian distance between the i-th pair of aligned residues,

*D*_0_(*L*) = 1.24. (*L *- 15)^0.33 ^- 1.8 is an *L*-dependant normalization factor.

The quality of each model generated by Modeller was predicted using the atomic distance dependant potentials DFIRE [32] and DOPE [33], and the knowledge-based potential ProQres which is derived from statistical distributions of atomic contacts, residue contacts, surface accessibility and secondary structure classes [34]. The individual evaluations obtained from DOPE, DFIRE and ProQres were then linearly combined yielding a composite score called SC3. The predictive accuracy of this score SC3 was optimized by maximizing the correlation between SC3 and the native versus model RMSD over a set of known knottin structures using a systematic grid search over the 3 DOPE, DFIRE and ProQres weighting factors. The model with the best SC3 score was selected and assessed by calculating its RMSD and TMS scores relatively to the actual native structure of the knottin query.

The models were also evaluated using free energy calculations based on molecular mechanics and empirical solvation energies using the MM_GBSA script from the Amber suite [35].

### Model refinement

1. LOOPM: After the homology modeling procedure, the best model was selected according to the evaluation score SC3 and all atoms but its first loop were frozen. 5 new query models are then obtained by ab initio modeling of the free loop using Modeller. All loops of the best model constructed so far according to SC3 were refined in turn following the same procedure.

2. LOOPY: The same refinement procedure as LOOPM was followed except that all loops were modeled using the Loopy prediction program [36].

3. LOOPH: The last refinement procedure consisted in successive local homology modeling restricted to each individual loop of the obtained knottin model. For each knottin loop of the best model produced so far according to SC3, the best template was selected according to the RMS criterion calculated over the given knottin loop only. The selected knottin loop template was then used to locally remodel the given query loop using Modeller.

## Results

### Knottin homology distribution

**Figure 2:**
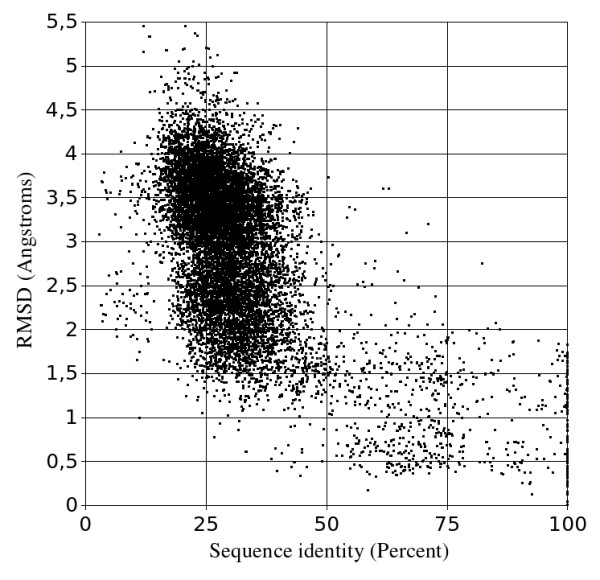
**Main chain RMSD versus sequence identity percentage for all knottin structure pairs from the PDB**.

**Figure 3:**
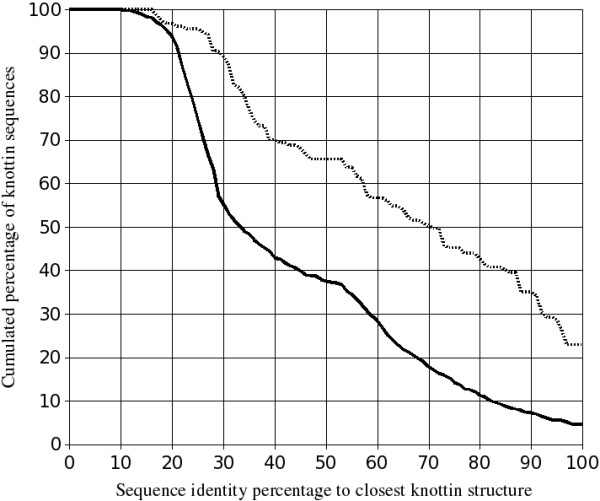
**Cumulated percentage of knottin sequences which share with their closest homologs with known structure a sequence identity percentage above the value indicated on the horizontal axis**. The continuous black line corresponds to the comparison of the 1466 sequences (all sequences but templates) against the 155 templates. The dashed black line above the black one corresponds to the comparison of each of the 155 templates sequence against the 154 other templates.

Figures 2 and 3 display sequence identity distributions over the whole knottin data set. Figure 2 indicates that the vast majority of known structure pairs share between 15% and 40% sequence identity (87% of all pairs) and 1.5 to 4.5 Å backbone deviation after geometrical superposition (90% of all pairs). This low level of average similarity clearly demonstrates the sequential and structural variability of the knottin superfamily. Knottins are indeed very diverse small proteins and the structural core of the whole family is actually limited to a few residues around the three knotted disulfide bridges.

We think that the tiny size of the conserved knottin core associated with the high degree of loop variability could explain the poor correlation between the sequence identity and the structural deviation. One should however note that the degradation of this correlation arises mainly below 40% sequence identity which corresponds anyway to low sequence conservation levels and then to significant structural variations in any protein family. This tendency is probably just amplified in knottins because of a smaller ratio between the size of the conserved structural core and the size of the exposed variable loops.

Figure 3 shows that half the knottin sequences share more than 33% sequence identity with their closest known structure, which is usually considered as a minimal threshold for homology modeling while the other half of knottin sequences will require a more challenging modeling at the low sequence identity level usually called the "twilight zone". However, knottins are specific miniproteins sharing a remarkably well-conserved cystine knot. The knotted cysteines are therefore expected to provide safe anchors that can be relied upon for sequence-structure alignments, hopefully allowing accurate modeling even at very low sequence identity. Nevertheless, a significant part of knottin structures is made of loops which are more difficult to predict than protein cores [37].

The comparison of both distributions on figure 3 also shows that the templates are, on average, more homologous to each other than the sequences are close to the templates. We expect this tendency to occur for many protein families since, unfortunately, not all homologous sequence clusters have one experimental structure known yet, and also because the PDB entries often correspond to different experimental structures of the same protein. For this reason, our modeling tests were made at various levels of allowed homology between query and templates (10%, 20%, 30%, 40% and 50% sequence identity).

### Template selection and alignment

**Figure 4:**
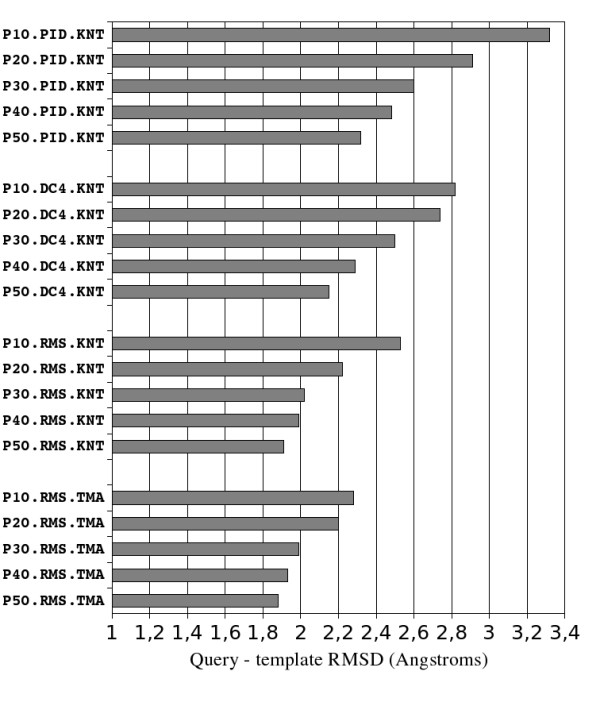
**Median query - template main chain RMSD for different template selection and alignment methods**. Each test is described by three concatenated fields on the vertical axis: 1^st ^field indicates the maximum allowed query - template sequence identity percentage, 2^nd ^field indicates which template selection criterion was used (PID, DC4 or RMS) and 3^rd ^field indicates with query - templates alignment method was used (KNT or TMA).

Figure 4 displays the median RMSD between the native knottin query and the 10 best structural templates selected according to different criteria. RMSD improves as templates are selected using the DC4 criterion rather than PID, and RMSD further improves when the criterion RMS is used. RMSD further improves when the template sequence are multiply aligned using TMA rather than KNT. The overall gain in RMSD between the worst and best selection method is high, from 1.08 to 0.44 Å median RMSD improvements when selected templates share less than respectively 10% to 50% sequence identity with query knottin. As explained in the following section, the quality of the best model built using Modeller is directly related to this template RMSD reduction.

Analysis of figure 4 shows that:

1. A careful selection of adequate template structures is important for high quality modeling as indicated by the significant RMSD reduction obtained by refining the selection criterion.

2. The PID criterion is not the optimal template selection method. The sequence identity percentage is a poor indicator of the actual structural similarity between two proteins. The weakness of PID is particularly clear in the context of knottins which form a widespread family and often require modeling at a low sequence identity.

3. Using sequence constraints derived from the analysis of all knottin folds can significantly reduce the average RMSD between the query structure and the selected templates. In the case of knottins, a hierarchical classification tree of all knottins guided by RMSD after pairwise structure superimposition has exhibited two sequential features, not included in the classical PID criterion, but that are directly correlated with the RMSD between knottin structures: (i) the length of each loop between knotted cysteines (from which was derived the criterion RMS), and (ii) the position of cysteine IV (from which was derived the criterion DC4).

4. Furthermore, the average RMSD between query knottins and their corresponding template structures can be significantly reduced when the query-templates sequence alignment is improved by using an appropriate alignment method. In the case of knottins, the Knoter1D and Knoter3D methods initially developed to align the knotted cysteines of knottins resulted in loop alignments that could be improved by the TM-align program which covers all core and loop residues for structural superposition.

### Model accuracy

**Figure 5:**
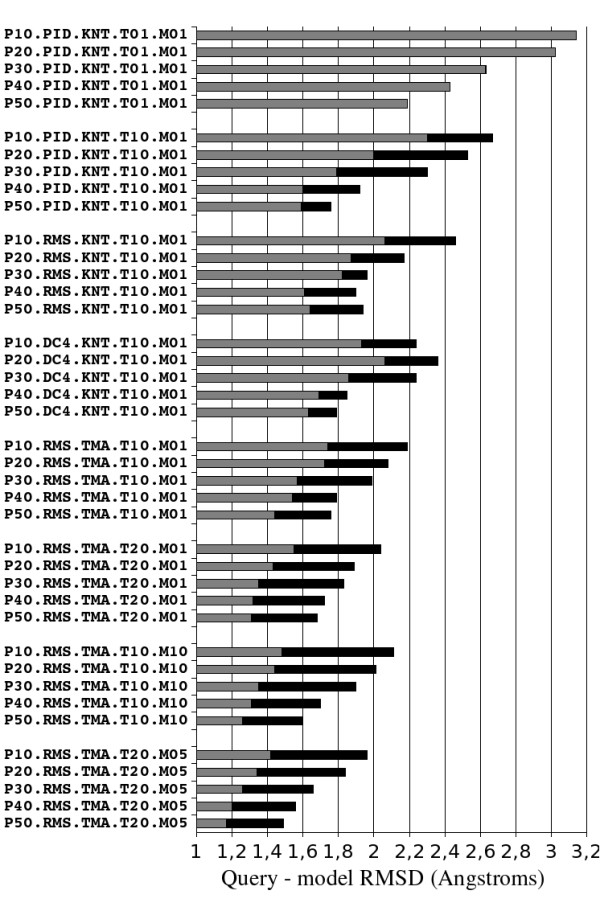
**Median query - model main chain RMSD for different modeling methods**. The grey end of each horizontal bar indicates the RMSD of the closest model to native structure while the black end of the bar indicates the RMSD of the best model according to SC3. Each test is described by five concatenated fields on the vertical axis: 1^st ^field indicates the maximum allowed query - template sequence identity percentage, 2^nd ^field indicates which template selection criterion was used (PID, DC4 or RMS) and 3^rd ^field indicates with query - templates alignment method was used (KNT or TMA), 4^th ^field indicates how many templates were used, 5^th ^field indicates how many models were generated at each Modeller run.

Figure 5 displays the median RMSD between native knottin queries and their corresponding best model built using Modeller and selected using the optimal linear combination of evaluation score SC3. As in figure 4, the median query - model RMSD is improving as templates are selected using 1) PID, 2) DC4, 3) RMS criteria. RMSD is further improved when the template sequences are multiply aligned using TMA rather than KNT. RMSD is also reduced when more templates are selected and when more models are produced by Modeller. The overall gain between the worst (PID.KNT.T01.M01) and best (RMS.TMA.T20.M05) modeling procedures varies from 1.18 Å to 0.70 Å median RMSD improvement when the selected templates share less than respectively 10% to 50% sequence identity with the query knottin. These gains in query/model RMSD are slightly higher than those observed in query/template RMSD (Figure 4). This spectacular model improvement indicates that the basic but frequently used modeling procedure using one template selected according to the percent identity relatively to the query sequence is far from optimal and could be greatly improved by combining multiple structural templates and by optimizing selections and alignments.

The best median query/model RMSDs are obtained by selecting 20 templates according to the RMS criterion, aligning them with the query sequence using the TMA algorithm, and producing 5 models at each Modeller run (Figure 5). With this modeling procedure, the median query/model RMSDs are 1.96 Å and 1.49 Å when the selected templates share less than 10% and 50% sequence identity with query knottin, respectively. The accuracy of the resulting models must be compared with the RMSDs observed between conformers within single NMR knottin structures in the PDB. The calculated average mean and maximum RMSDs between such conformers are 0.79 and 1.38 Å, respectively. At a 50% level of sequence identity, the accuracy of the models (1.49 Å RMSD) is therefore very close to the average maximum variation between NMR conformers (1.38 Å). It should be also noted that, on figure 2, even at 100% sequence identity experimental knottin structures can diverge by more than 1.8 Å. Native protein flexibility, domain or external interactions, and experimental errors may explain these variations. These comparisons strongly suggest that our procedure is close to the optimum of what can be achieved computationally in knottin modeling.

Another interesting observation is that the model versus native main chain RMSD decreases as the number of selected templates per knottin query increases. That multiple templates complement each other could be explained by the observation that the conserved core across all knottins is mainly limited to few residues nearby the three knotted disulfide bridges while the inter-cysteine knottin loops have very diverse conformations. It is therefore often impossible to find one single template carrying inter-cysteine loops compatible with all query loops. As a result, selecting several structural templates, which individually cover the conformations of each query loop, may be required. Actually, the exact number of templates selected to build the model with lowest RMSD relatively to the native query structure is randomly varying from one to the maximum number of allowed templates. This variation of the optimal number of templates confirms that the geometrical constraints inferred from the different structures are frequently complementary.

The same statistical analysis was done using TMS instead of RMSD as structural similarity criterion. The different modeling procedures were ranked using TMS in the same order as RMSD. Considering knottins as a small conserved core of knotted cysteines connected by flexible loops of varying sizes, we anticipated TMS to be a more accurate measure of the knottin core conservation since TMS reduces the weight of loop displacements. Apparently, this is not case and the RMSD produces measures comparable to TMS, indicating that core and loop variations in knottins are more connected than what we predicted.

The 3 knotted disulfide bridges and the 5 or 9 80% conserved H-bonds depending on the position of cysteine IV can be observed in all generated models. When the restraints on the 80% conserved hydrogen bonds are removed from the Modeller script, only insignificant variation in median query-model main chain RMSD (0.04 Å) is observed, but the network of conserved hydrogen bonds is then usually degraded and the computed models frequently miss the main chain bonds present in most experimental knottin structures. Furthermore, the packing quality of the models is clearly improved at any homology level by restraining the conserved hydrogen bonds, yielding an average 12.7% increase of the Errat scores of the hydrogen bond constrained knottin models over the non constrained ones. Although the improvement is not measurable by a gain in query - model RMSD accuracy, it is important to note that these additional restraints guide the generated models towards better structural packing and conformations more consistent with the knottin consensus fold. This result indicates that useful geometrical restraints can be inferred from the comparative analysis of all experimental structures related the query protein.

**Figure 6:**
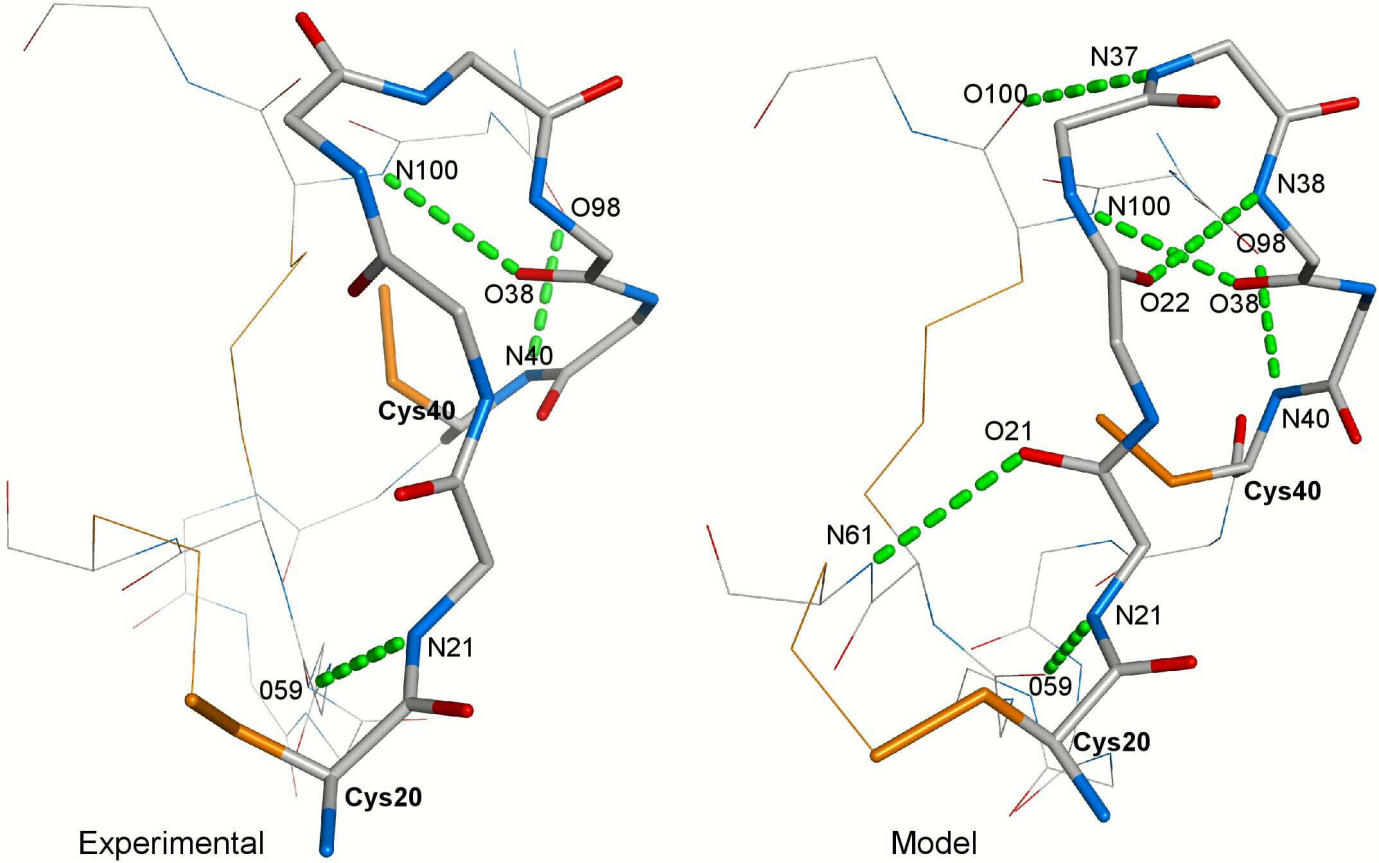
**Experimental and model structures of GsMTx-4**. The CysI-CysII loop is shown as sticks. The protein segments interacting with the loop are shown as lines. Consensus hydrogen bonds are labeled and displayed as dashed green sticks. Nitrogen atoms, oxygen atoms, and cysteine side chains are shown in blue, red and orange, respectively.

Figure 6 displays the CysI-CysII loop (loop 'a') of the experimental structure of the spider toxin GsMTx-4 (PDB:1LU8) and the corresponding model with the best SC3 score (RMSD 1.21 Å). Clearly, only small deviations of loop 'a' conformation are necessary in the model to accommodate six consensus hydrogen bonds when compared to the experimental loop involved in only three hydrogen bonds.

**Figure 7:**
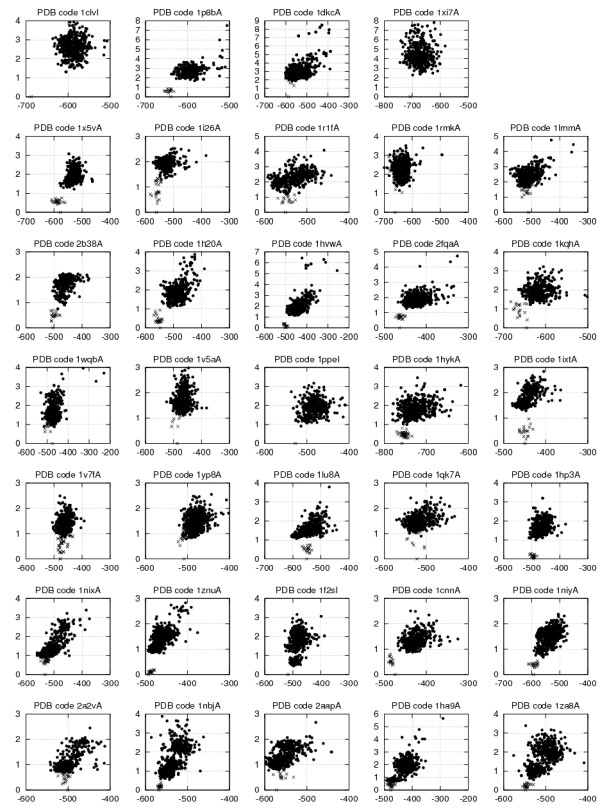
**Model - native main chain RMSD (vertical axis) versus model SC3 score (horizontal) for each of the 34 knottins queries over all structural models generated with the modeling method RMS.TMA.T20.M05**.

Figure 7 shows the correlation between the native versus model backbone RMSD and the combined score SC3 of all models constructed for each of the 34 knottin queries from the test set. To facilitate visual comparisons, the knottin queries were sorted in a top-down order from the worst to the best produced models. SC3 is usually well correlated to RMSD when the best models are close to the native structure, with RMSD typically below 1.5 Å, while SC3 is often not a good accuracy predictor when the best models have higher RMSD relatively to the native structure.

**Figure 8:**
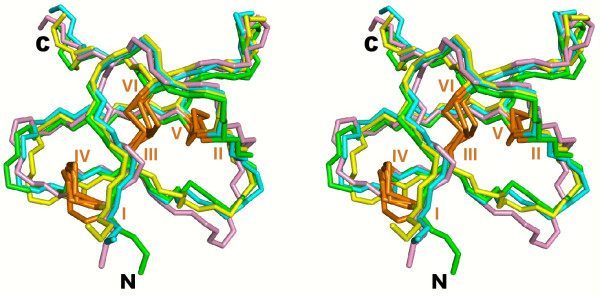
**Stereoview of experimental and model structures of hainantoxin-4**. Green, the NMR structure (PDB ID: 1niy); cyan, the model with the lowest RMSD; yellow, the model with the lowest SC3 score; violet, the model with the lowest SC3 score obtained from templates with less than 10% sequence identity with hainantoxin-4. Disulfide bridges are labeled and shown in orange.

The experimental knottin structures from the test set were also evaluated using SC3 and the RMSD of each NMR conformer from the PDB file relatively to the first one were calculated. These evaluations, displayed as crosses in Figure 7, show that:

1. Although the structures from the PDB files have on average better SC3 scores than the corresponding models constructed by our procedure, the best models usually display SC3 scores close to or even better than the best experimental structures. This scoring similarity suggests that our procedure achieves a sufficient conformational sampling to build knottin models that are energetically close to the optimum measured on the native structures. As an example, the hydrogen bond network in the GsMTx-4 model shown in Figure 6 is likely responsible, at least in part, for the better scores displayed by many models when compared to the NMR structure (see Figure 7, 1lu8A). In contrast, the experimental structure of hainantoxin-4 (PDB:1NIY) displays all knottin consensus hydrogen bonds and gives good SC3 scores. Figure 8 shows a superimposition of the experimental structure and of structures modelled from templates at different sequence identities. The best scoring model built from templates with sequence identities below 10% (violet structure in Figure 8) is still reasonably accurate with an RMSD to native of 1.22 Å).

2. The RMSD between experimental conformers for the same PDB entry are often comparable to RMSDs between the best predicted models and the native structures, indicating that the best models are consistent with the flexibility observed in experimental structures. In other cases, when the inter NMR RMSD is smaller than the model to native RMSD, one can wonder which of the model or of the NMR conformations were flawed. When the inter NMR RMSD is always below 0.5 Å, one can suspect that, except for the shortest knottins, the loop conformations of the corresponding NMR structures were too constrained or not sufficiently sampled to correctly represent the natural flexibility of the longest and exposed amino acid segments. This may arise from standard NMR refinements that simultaneously apply all NMR constraints and do not take into account the NMR time scale averaging, thus resulting in all conformers lying near an average conformation rather than really sampling the available conformational space.

### Optimization of the evaluation score SC3

**Figure 9:**
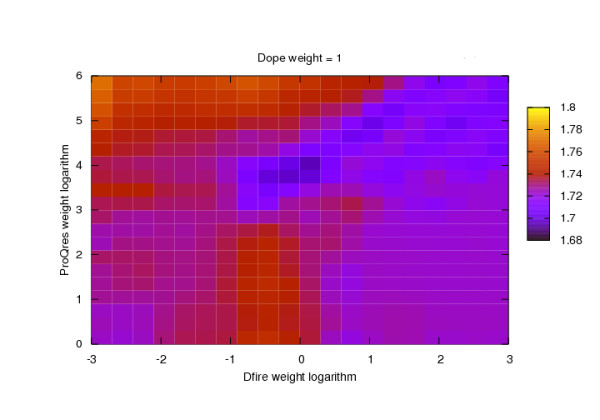
**Color coded median model - native main chain RMSD depending on the logarithm of the weights used for linearly combining DFIRE and ProQres in the composite score SC3 (DOPE weight is fixed to value 1)**.

The scores DOPE, DFIRE and ProQres were linearly combined yielding a composite evaluation score whose weights were optimized by grid search. Figure 9 displays the variation of the average RMSD between the native structure and the best evaluated model depending on DFIRE and ProQres weight logarithms (DOPE weight is fixed to value 1). Models were obtained from the best modelling procedure RMS.TMA.T20.M05. From Figure 9, Dope = 1, DFIRE = 1 and ProQres = 49 are the optimal weights for linear combination yielding an average native - model RMSD of 1.68 Å. This optimal linear weight combination was used for all the evaluations displayed in figures 5 and 8. The performances of each score DOPE, DFIRE and ProQres used individually were respectively 1.72, 1.72 and 1.79 Å. The improvement due to their linear combination is therefore 0.04 Å only, indicating a small complementarity of the different evaluation scores.

### Loop refinement

**Figure 10:**
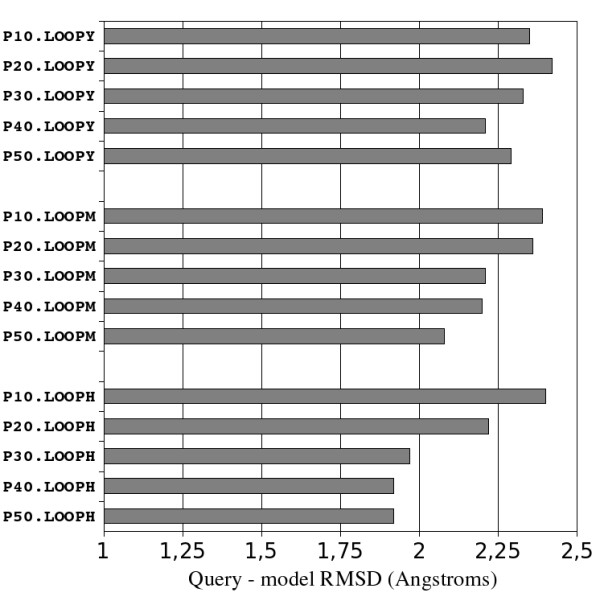
**Median query - model main chain RMSD for the loop refinement methods LOOPM, LOOPY and LOOPH**.

As indicated in figure 10, the 3 loop refinement procedures we have tested failed to improve the accuracy of the best homology models. The median query/model RMSD increases are around 0.4 and 0.4 - 0.7 Å at 10% and 50% sequence identity levels, respectively (compare Figure 10 and Figure 5, bottom). It is difficult to interpret the reason of this model degradation. One possible explanation could be that the loops are refined individually while freezing the rest of the protein structure. Incorrect loop anchor orientations or wrongly placed interacting loops could then force the refined loop to explore a wrong conformational space yielding a degradation of the query/model RMSD. To solve this problem, we tried to extend the loop boundaries at varying sequential distances of the knotted cysteines but this did not improve the model accuracies significantly (data not shown). RMSD increase could also be related to the incremental nature of the refinement procedure: if one loop is wrongly refined and accepted by SC3 as an improved model then all subsequent loop refinements will be done in a wrong structural context and then biased toward incorrect orientations. We designed the LOOPH procedure to address this latter issue: the best local templates were selected for each loop and an aggregation of these local templates/loop alignments was built to let Modeller make a global refinement of the best model obtained so far by freezing the knotted core and using the best local templates to refine all loops at the same time. The accuracy of the models were still degraded using the LOOPH refinement procedure indicating that freezing the loop anchors induces too strong constraints on the conformational space that can be explored by Modeller.

### Minimization of the model energy

**Figure 11:**
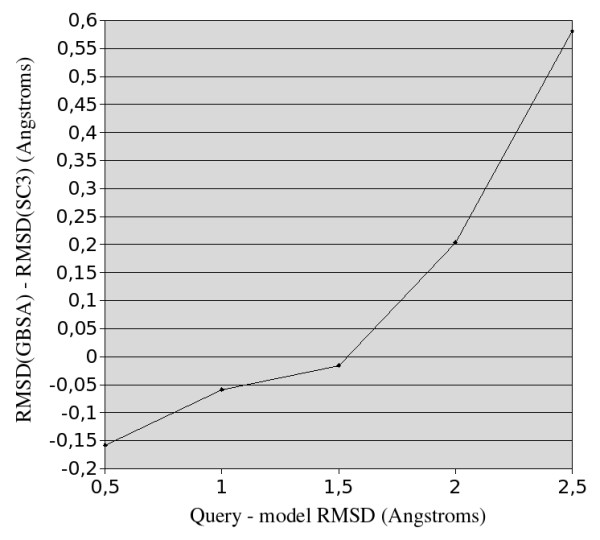
**Median model - native main chain RMSD variation when scoring the models with MM_GBSA instead of SC3 versus the model - native main chain RMSD**.

Figure 11 displays variations of the model - native structure RMSDs when the models are energy minimized using the Amber suite then selected using the MM_GBSA energy as the evaluation criterion. A recent study has shown that energy minimization with implicit solvent (GBSA) provides greater improvement for some proteins than with a knowledge based potential [38]. Unfortunately, on our data set, while requiring more computing time, this refinement and evaluation method suffers globally from a slight loss in accuracy compared to the SC3 criterion, resulting in a RMSD variation below 0.1 Å between the two criteria. It is however worth noting that the MM_GBSA criterion is slightly better than SC3 when models are close to the native structure (RMSD < 1.5 Å)but worse than SC3 when models are farther from the native structure (Figure 11). This result tends to indicate that physics-based force fields with implicit solvation (MM_GBSA) are better in assessing quality of models close to the native state while knowledge-based potentials are more accurate predictors when deformations are higher. This tendency is consistent with the preferential uses of statistical potentials for threading or folding prediction at low sequence identity and of physics-based force fields for the refinement of models close to native conformations. This dichotomy suggests that model selection could be improved if we could predict which criterion to use, either MM_GBSA for models closer than ~1.5 Å to native structure or SC3 for more distant models. However, such a close - distant model classifier would need to be quite accurate since misclassifications would rapidly cancel the small gain obtained using MM_GBSA for close models.

### Model database and server

The 1621 known knottin sequences were extracted from the latest release of the KNOTTIN database [1]. A structural model of each knottin sequence was built using the optimized procedure detailed above: 20 templates were selected according the TMS criterion and without restriction on the query versus template sequence identity. These templates were multiply aligned with the query sequence using the TMA procedure. Then, using from 1 to 20 aligned templates, 5 structural models of the query were generated at each Modeller run after imposing appropriate constraints on the knotted disulfide bridges and the 80% conserved hydrogen bonds. The 20 Modeller runs resulted in 100 structural models per query which were sorted according to the SC3 criterion. Finally, the energy of the best model was minimized using the sander program of the Amber package. Restraints were applied on the backbone atoms to avoid large deviations from the initial model and the GBSA implicit solvation scheme was used. Further difficulties arise when attempting to automatically model large data sets. Since several knottins are macrocyclic, i.e. the N- and C- termini are connected through a regular peptide bond, potentially cyclic knottins (mainly cyclotides) were tentatively modeled as such according to the annotation available in the KNOTTIN database [3]. In the latter database, the cyclic feature was assessed by manually analyzing the N- and C-termini for the presence of a cyclization site. Moreover, a large number of knottins display additional disulfide bridges that supplement the 3 disulfides forming the cystine knot. These additional bridges were only imposed in the models when there was no ambiguity regarding cysteine connectivity. In any case, when residues at standard positions 82 and 98 were cysteines, a disulfide bridge was always imposed whatever the total number of cysteines, since this bridge has been frequently observed in experimental structures (see Figure 1). Finally, except for knottins with known 3D structure, the resulting knottin structural models are now available from the "Sequence" section of the KNOTTIN database server at URL http://knottin.cbs.cnrs.fr[3]. New models will be added as novel sequences are discovered and incorporated in the Knottin database. By comparing the knottin sequence identity distribution (Figure 3) with the expected model accuracy (Figure 5), the average model versus native structure RMSD over all knottin sequences can be estimated between 1.6 and 1.7 Å which should be a sufficient accuracy for many applications.

The homology modeling procedure has also been integrated into the protein analysis toolkit PAT accessible at http://pat.cbs.cnrs.fr[39] as an independent structural prediction module called Knoter1D3D. The whole processing for one knottin structure prediction requires one minute to one hour on this server. This processing time depends linearly on the product of the chosen maximal number of 3D templates and of the number of models generated per Modeller run. The best resulting knottin model is saved as PDB formatted data and is accessible from the PAT web session manager. By this way, knottin data can be further analysed by interactive data transfer to other analysis tools available in the PAT processing environment.

## Discussion

### Modeling at low sequence identity can be improved by a structural analysis of template clusters

Although continuous improvements in the accuracy of protein modeling techniques have been achieved over the last years, structural predictions at low sequence identity still remain difficult. In this work, we have shown that the optimal use of the structural information available from all members of the query family can lead to notable model accuracy and quality gains, even when the closest templates share less than 20% sequence identity with the protein query. For example, the DC4 criterion, which was shown to improve template selection, could be directly derived from the analysis of the disulfide bridges and hydrogen bonds conservation over all knottin structures. Using a hierarchical classification of all knottin structures, we could evidence a direct influence of the position of cysteine IV onto the main chain hydrogen bond network. Such structural information can be easily translated into a sequence constraint by adding, to the PID criterion, a penalty when template and query cysteine IV cannot be aligned. Benchmarks on our knottin test set showed that this modified DC4 criterion achieves a better template selection than PID alone. This example demonstrates that generic modeling approaches applicable to any protein are too general for optimally modeling a specific protein family because they are not able to delineate precisely the structural features conserved over related protein subsets. Furthermore, in our work, the conserved hydrogen bonds derived from structure superimposition and clustering were used as restraints to force the models to conform to the 80% consensus hydrogen bonding observed over the whole knottin family or a subset of it. This is useful because not all templates satisfy the consensus hydrogen bonds, most likely because hydrogen bonds cannot always be directly inferred from NMR data. Consequently correct hydrogen bonding, especially in solvent exposed areas, strongly depend on the structure calculation and refinement methods. Moreover, the use of multiple templates in the modeling (see below) may result in averaging and, locally, to the loss or deformation of specific hydrogen bonds. Nevertheless, improvements from such specific constraints cannot be easily quantified by RMSD reductions but rather by a better organization and conformation of the main chain, i.e. better quality models as demonstrated by increased Errat scores at any homology levels.

### Modeling at low sequence identity can be improved by combining more templates

Another important result of this work was the important reduction of query - model RMSD obtained by combining multiple structural templates for modeling one query. For the best modeling procedure RMS.TMA.M05, the query - model main chain RMSD reduction was on average 0.38 Å when SC3 was used as model assessor and when up to 20 templates were used instead of only one. This result is consistent with what has been observed recently on more diverse structure sets using Modeller as model generator and ProQ as model assessor [40]. This improvement might have been reinforced for knottins because the large sequence diversity, the tiny conserved core and the high structural loop variability often imposed the use of many templates to cover the conformational space of each query loop. Using multiple templates extends the conformational space explored by the models while the SC3 filter is sufficiently accurate to select, on average, better models as their number increases. Actually, the number of combined templates resulting in the most accurate model was varying between 1 and the maximum allowed number 20 over the different knottin queries with a mean value near 10. The optimal models were therefore usually obtained from more than one template, thereby indicating that even the more distant templates help to better capture the target fold.

### Modeling at low sequence identity can be improved by procedural optimization

Modeling at low sequence identity requires a succession of processing steps which can be combined in many ways. The knottin template and model accuracies display important variations when different modeling procedures and parameters are chosen as can be seen from figures 4 and 5. In particular, it can be observed that a basic modeling procedure based on a unique template per query is far from optimal, particularly when the templates are weakly homologous to the query. This performance variation stresses the importance of systematically optimizing each processing step, of exploiting in each step the structural constraints specific to the query family and of measuring the impact of each modification on a relevant test set. Using the modeling procedure optimized on knottins, it is interesting to note that the resulting query - model RMSD was 0.14 Å below the smallest query - template RMSD on average (data not shown). This result is significant since building models closer to native experimental structures than the templates used to build them is usually considered as the major challenge of homology modeling for years to come.

### Best models could be improved by energy minimization with implicit solvent

Implicit solvation schemes can help classical molecular mechanics force fields to better refine and evaluate protein structural models [38,41]. We observed a similar impact on our data set when MM_GBSA was used for refining models close to native fold, but an opposite impact when the models deviated from native for more than ~1.5 Å. This trend is consistent with the intuitive observation that energy minimization can be efficient only if the initial conformation lies within the energy basin corresponding to the native minimum. When this condition is met, implicit solvent improves the minimization and the evaluation obtained from the physics-based force fields by refining the assessment of the residues exposed to solvent and by smoothing the rugged energy landscape thereby helping to escape local minima. An important and positive side effect of energy minimization is to optimize the hydrogen-bonding network and to remove any steric clash that could arise when combining incompatible restraints from different templates. Unfortunately, the degradation observed for the models with deviation from native state higher than 1.5 Å was not compensated on average by the improvement obtained on the closer models. Recently, notable progress was made on the structural evaluation and correlation coefficients above 0.9 between the model scores and the model - native main chain deviation were reported [42]. If such a reliable model assessor could be designed for knottins, then energy minimization with implicit solvent could be profitably focused on the best predicted models only.

### How to model knottin loops

A correct modeling of knottin loops is important since loops constitute a major fraction of the knottin structures. Unfortunately, sequential RMSD distribution indicates that the knottin cores are usually accurately modeled while the major fraction of query - model deviation is concentrated in the loops. Our various attempts to refine knottin loops failed probably because the explored conformational space was too narrow and because the evaluation criterion SC3 was unable to correctly assess these irregular and solvent exposed segments. We showed in previous studies how context-dependent potentials can accurately evaluate the compatibility of a given amino acid with very specific structural environments [43,44]. To improve the structural evaluation of the knottin loops, we have developed knowledge-based potentials dependent on each loop length and anchor geometry. The potentials were calculated as follows: all loops with a number of amino acids identical to the model loop and a relative orientation of the anchoring residues similar to the model loop are extracted from the PDB and a statistical scoring profile is then derived from the positional amino acid and conformation frequencies observed in these selected loops. Such statistical profile reflects specifically the conformational propensities of any amino acid segment locally grafted on the considered model. However, the incorporation of these loop dependant potentials into the model evaluation score SC3 did not improve its accuracy. Nevertheless, many issues remain to be explored about these potentials such as how to normalize the potentials for comparing different loop anchors or how fine should be the loop sampling for a given sequence length and anchoring geometry. In combination with a rapid loop generator such as Loopy [36], such loop-specific potentials are promising tools for adding context specific information and guiding the exploration of the loop conformational space.

## Conclusion

In this work, we have optimized a modeling pipeline to build 3D models of proteins with the knottin scaffold. The fully automatic and optimized process allowed us to generate satisfactory models for the 1621 known knottin sequences which open the way toward applications requiring intermediate resolution atomic coordinates. Applications based on the knottin models are beyond the scope of this article. Nevertheless, we expect that the exhaustive knowledge of all knottin structures will be useful for refining their classification since sequence identities are sometimes so low that evolutionary relationships can be very ambiguous. Other major applications of knottin models might be the prediction of interaction sites for which many approaches with diverse levels of reliability have been developed [45-47]. It would be interesting to apply these tools for delineating the few functionally critical residues and their 3D signatures, or for predicting non-continuous epitopes [45-47]. It has been shown also that antimicrobial peptides often interact with membranes through non-specific sites made of a combination of hydrophobic surfaces and positively charged clusters [48,49]. Such features could be systematically searched in knottin 3D models to suggest new potential drug leads.

Although this work is specific to a particular small disulfide-rich scaffold, we expect that the improvements obtained here could be transposed to larger and more representative protein family sets. Apart from the computational time which will be higher for larger proteins, all methods described here are fully automated and processing other families should be relatively easy. Protein families with large structural variability should benefit most from the improved template selection and alignment methods, from the combined use of varying numbers of templates, and from the refined model evaluation scores. Furthermore, the structure analyses of the related templates that led to disulfide and hydrogen bond restraints could be applied to other families and even generalized to other structural features such as main chain conformation or amino acid interactions. This type of analysis method could even be refined by automatically delineating template subsets sharing discriminative structural features and corresponding to particular branching nodes in their classification tree. In particular, such discriminant analyses could permit the definition of geometrical restraints specific to different interaction sites in the case of protein superfamilies which cover several functions and binding modes.

## Authors' contributions

JG and LC designed the project, interpreted the results, implemented the internet server modules and written the manuscript. JG implemented the modeling procedure, processed data and computed result statistics. All authors read and approved the final manuscript.

## Aknowledgements

We are grateful to Conan Wang and David Craik for a preliminary study and helpful discussions.
